# Cytotoxicity of Cold Ceramic Compared with MTA and IRM

**Published:** 2009-07-06

**Authors:** Mohammad Ali Mozayeni, Amin Salem Milani, Laleh Alim Marvasti, Fatemeh Mashadi Abbas, Seyed Jalil Modaresi

**Affiliations:** 1 *Department of Endodontics, Iranian Center for Endodontic Research, Dental Research Center, Dental School, Shahid Beheshti University of Medical Sciences, Tehran, Iran.*; 2 *Department of Endodontics, Dental School, Tabriz University of Medical Sciences, Tabriz, Iran.*; 3 *General Practitioner and Researcher, EDO and Iranian Center for Endodontic research, Dental School, Shahid Beheshti University of Medical Sciences, Tehran, Iran.*; 4 *Department of Oral and Maxillofacial Pathology, Dental School, Shahid Beheshti University of Medical Sciences, Tehran, Iran.*; 5 *Department of Endodontics, Dental School, Yazd University of Medical Sciences, Yazd, Iran.*

**Keywords:** Cold ceramic, Cytotoxicity, Fibroblast, Intermediate restorative material, MTA, New endodontic material, Root end filling

## Abstract

**INTRODUCTION:** Biocompatibility is a desirable feature for root-end filling materials. In this study we aimed to compare a new material called cold ceramic (CC) with intermediate restorative material (IRM) and mineral trioxide aggregate (MTA) using Methyl-tetrazolium bromide (MTT) assay.

**MATERIALS AND METHODS:** The materials were tested in fresh and set states: (n=108). The cytotoxicity was compared using L929 fibroblasts as an indicator; tested materials were eluted with culture medium according to ISO: 109935 standard. Distilled water and culture medium served as positive and negative controls, respectively (n=36). The results were evaluated at 1, 24 hours and 7 days. Data were statistically analyzed by one-way ANOVA for each time interval and material status and t-tests.

**RESULTS:** The cytotoxicity of the tested materials were statistically different at the various time intervals (P<0.001). IRM was the most cytotoxic root-end filling material (P<0.001), MTA demonstrated the least cytotoxicity followed by CC.

**CONCLUSION:** Despite displaying the greatest cytotoxicity, IRM is approved by the American Food and Drug Administration (FDA). Cold ceramic had significantly lower cytotoxicity compared to IRM, in all but one subgroup. Further investigations are required to assess the clinical applicability of this novel material.

## INTRODUCTION

Though extraction and implant placements have become more popular with success rates of 98.4% after 15-57 months; research has also shown excellent success rates (99.3% after 18-59 months) for root canal therapy (1) and surgical endodontics (91.5% after 5-7 years) (2). Surgical endodontics is often performed as a last resort, after considering or carrying out root canal therapy (RCT) or re-RCT (3). There are many reasons why apical surgery is adopted, for example complex and difficult root canal system, strategic or esthetic prosthesis, iatrogenic operative blockages or overfills (4). Root-end filling material should have particular characteristics which have been well documented *i.e.* insolubility, biocom-patibility and cementogenesis, sealing ability*,* dimensional stability, moisture imperviousness, radiopacity and ease of handling (5).

The close proximity of periodontal tissues to the root apex and their extensive blood supply make biocompatibility very desirable feature for root-end filling materials (3,5,6).

MTA is materials that can inducecementogenesis (7) due to its high biocompatibility and sealing ability (8).

MTA has been extensively researched and compared to IRM *in-vitro *(5,9) and *in-vivo *(10)*.* A clinical trial by Chong *et al*. demonstrated that after 12 and 24 months, MTA had greater success rates than IRM, though statistical significant difference was not found. MTA biocompatibility has been extensively assessed by SEM and enzyme assays (11); an investigation which had been carried out on primary and established cell lines focused on the effect of two types of MTA for perforation repair as well as GP, amalgam, GICs and super EBA; this study found that ProRoot MTA and Angelus MTA were superior nontoxic agents (12).

Cytotoxicity has also been investigated for MTA on human PDL cells (13); white and grey MTA on human osteoblasts (14) and ProRoot MTA on human fibroblast PDL cells (15); most of the articles concluded that MTA has the least cytotoxicity (13-16).

MTA has various uses such as pulpotomy of primary and permanent teeth (17) as well as pulp capping, perforation repair and root-end restorations (18,19).

A possible low cytotoxicity of CC will also suggest whether CC may also have similar uses (20).

*In vitro* and* ex vivo* tests are essentially a form of screening (21); materials that demonstrate high cytotoxicity can be precluded from more expensive and complex *in vivo* investigations (10,22). The regeneration of periodontal and periradicular tissues around the root-end restorations would be the ideal biological response (9,19).

L929 Fibroblasts are the most commonly used standardized established cell line for assessing cytotoxicity as the results are more likely to be reproducible (13,23). International Standards Organization (ISO) has recommended preliminary cytotoxic screening (ISO 7405) with these cells (13,37).

We aimed to assess the cytotoxicity of Cold Ceramic compared to established materials IRM and MTA.

## MATERIALS AND METHODS

The study protocol was approved by Shahid Beheshti University, Tehran, Ethical Committee.

***Study design***

This *ex vivo* study was designed to evaluate the cytotoxicity of three root-end filling materials on L929 mouse fibroblasts with MTT assay and Elisa reader. The groups were divided as follows: Group 1 consisted of IRM fresh and set, Group 2 consisted of MTA fresh and set, and Group 3 CC fresh and set. Each group was assessed at a) 1 hour (subgroup A, b) 24 hours (subgroup B), c) 7 days (subgroup C). Each group consisted of 36 samples and each subgroup of 12 samples each (total of 108). A further 36 samples were put aside for positive and negative controls (n=18). Distilled water and complete DMEM were used as the positive and negative controls, respectively, instead of extraction vehicle.

All samples were placed in incubator set at 37^°^C, 98% humidity and 5% CO_2_, and removed at the appropriate times (*i.e.* 1 hour, 24 hours or 7 days). Note that for the 7 day time span the extraction vehicles were replenished every 48 hours to sustain cell viability.

The L929 mouse fibroblasts (Pastor Institute, Tehran, Iran) were taken from solid form and de-solidified and washed out in tryspin (USA and GIBCO) 4 times.

Specimens with a vitality rate of over 95% were utilized for the investigation. The cells were then placed on a slide and counted with hemocytometer. A monolayer of cells totaling 6,000 was placed inside each well (plates contained 96 wells each) (Cellstar, Greinerbio-one). Three plates were used for materials, for each of the time intervals. The negative and positive controls also had 3 plates each. The culture mediums were placed in Dulbeccos’ modified Eagle’s medium, (DMED), (Life Technologies Inc Grand) fortified with 10% Fetal Bovine Serum (FBS) as well as penicillin antibiotic 100 IU/mL (Sigma USA) and streptomycin 100 µg/mL (Sigma, USA). The samples were kept at 37^o^C, 98% humidity and 5% carbon dioxide.

Root-end filling materials were prepared according to manufacturer’s and inventor’s instructions and mixed in an aseptic environment with a sterile instrument. Fresh subgroups were immediately placed in 3 mm thicknesses inside wells. The set subgroups were prepared in 3 mm thicknesses and then placed in 98% humidity and 37^o^C under UV light for 24 hours. The extraction vehicle in our study contained complete DMED with normal saline (pH balanced) and the root-end filling material. After 10 minutes of preparing the subgroups, the 4 mL of culture mediums were added to experiment and control wells. Afterwards, the extractions were filtered with 0.22 µm syringe (Schjeicher and Schwell) to ensure sterile conditions were met.

For carrying out MTT assay, 5mg/mL methyl tetrazolium bromide salts (Sigma-Aldrich, USA) in phosphate buffer solution (PBS) were mixed with DMEM (ratio of 1:10). The extraction was first evacuated and 150 µL of this solution was added to each well. The material was then incubated for four hours in identical conditions (*i.e.* 37^o^C, 98% humidity and 5% CO_2_).

 The top layer of the solutions was removed and instead 150 µL of isopropananolic acid was added to each well. Next 100 µL was taken from each well and placed in the Elisa reader (Anthos 2020, Australia) microplates. Optical density had initially been collaborated with the negative control value to obtain an accurate comparison. Optical density was then assessed under a wavelength of 570 nm and a filter reference of 620 nm.

Two-way ANOVA test were performed. Independent t test and Tukey test were also used for pair comparison. Statistical significance was established at P<0.05.

## RESULTS

Table 1 indicates that the cell viability of Cold Ceramic equaled MTA and negative control in both set and fresh states after one hour. IRM demonstrated the lowest cell viability, significantly less than CC and MTA in both states. Set IRM had no cell viability (*i.e. *equalling positive control). After 24 hours, MTA had statistically greater cell viability than CC in both states (P<0.001); and CC demonstrated superior cell viability to IRM and positive control in both states (P<0.001). In subgroup C (7 days), fresh and set states demonstrated significantly different results (Table 1 and 2). Fresh CC demonstrated similar cytotoxicity to fresh IRM and positive control. The cell viability values for set CC, on the other hand, was greater than set IRM and equalled set MTA. We can conclude that negative control>(set MTA=set CC) and (set MTA=set CC)>(set IRM=positive control) and also, that negative control>fresh MTA> (fresh CC=fresh MTA=positive control).

The lowest recorded cytotoxicity was expressed by MTA in 5 of its subgroups, but only in one of CC subgroups (Table 2).

## DISCUSSION

In this study set and fresh material were analyzed to determine whether cytotoxicity resulted from setting process or the by the by-products the set material released. Most studies do not analyze materials after 7 days (21,24) as maintaining vitality for long durations requires extra care; in this study the extract medium was changed every 48 hours (25).

MTT is a simple and popular *in vitro *test but with certain limitations (26,27). The *ex vivo *environment does not replicate the biological response of the periapical tissues; *in vivo* tests would therefore be advisable if positive results were obtained from *ex vivo *tests.

MTA is steadily becoming the gold standard material in endodontics with which many new materials are compared (5,28).

In this study, only three subgroups of MTA demonstrated superior cell viability to CC: set and fresh MTA in subgroup B (24 hours) and fresh MTA in subgroup C (7 days). In all other subgroups CC and MTA had similar cell viability values suggesting that CC may be a viable option as a relatively biocompatible root end filling material. Cold Ceramic’s sealability has been compared with MTA introduced as a suitable alternative to MTA, in terms of sealability (29,30). In this study CC demonstrated competitive cell viability values when set; moreover, CC was consistently second or equal to MTA. A material that has repeatedly demonstrated low cytotoxicity (31,5). The biocompatibility of MTA has been commonly associated with their ability to release calcium ions as they set and the subsequent reaction with phosphorous to form hydroxyapatite crystals, facilitating healing (32). Camilleri *et al.* showed that fresh MTA has greater biocompatibility than its set state (33); the fresh form better emulates the clinical usage of this material in retrograde fillings. Our results however, show that there are no differences between set and fresh states except after 7 days (subgroup C) where set state is superior. In group C, fresh CC had significantly poorer cell viability compared to its set state; the fresh state statistically equaled IRM values and set state equaled set MTA (P>0.05) (Tables 1 and 2).

**Table 1:** Comparison of Cell viability values after 1, 24 hours and 7 days (subgroups A, B and C) among tested materials

Time	Subgroup	CC ^†^ (set and fresh)	IRM ^‡^ (set / fresh)	Positive control	Negative control
**One Hour (A)**	fresh IRM	freshCC>freshIRM^*^	__	freshIRM>control^*^	freshIRM>neg^^^^control^*^
	set IRM	setCC>setIRM^**^	__	setIRM=control	setIRM> neg control^**^
	fresh MTA^#^	freshMTA=freshCC	freshMTA>freshIRM^*^	freshMTA>control^*^	freshMTA=neg control
	set MTA	setMTA=setCC	setMTA>setIRM^**^	setMTA>control^*^	setMTA=neg control
	fresh CC	__	See above	setCC>control^*^	freshCC=neg control
	set CC	__	See above	freshCC>control^*^	setCC=neg control
**Twenty Four Hours (B)**	fresh IRM	freshCC>freshIRM^*^	__	freshIRM=control	freshIRM>neg^^^^control^*^
	set IRM	setCC>setIRM^*^	__	setIRM=control	setIRM> neg control^*^
	fresh MTA^*^	freshMTA>freshCC^*^	freshMTA>freshIRM^*^	freshMTA>control^*^	freshMTA>neg control^*^
	set MTA	setMTA>setCC^*^	setMTA>setIRM^*^	setMTA>control^*^	setMTA=neg control
	fresh CC	__	__	freshCC>control^**^	freshCC>neg control^*^
	set CC	__	__	setCC>control^*^	setCC>neg control^*^
**Seven Days (C)**	fresh IRM	freshCC=freshIRM	__	freshIRM=control	freshIRM>neg^^^^control^*^
	set IRM	setCC>setIRM^*^	__	setIRM=control	setIRM> neg control^*^
	fresh MTA^*^	freshMTA>freshCC^**^	freshMTA>freshIRM^*^	freshMTA>control^*^	freshMTA>neg control^*^
	set MTA	setMTA=setCC	setMTA>setIRM^*^	setMTA>control^*^	setMTA>neg control^*^
	fresh CC	__	__	freshCC=control	freshCC>neg control^*^
	set CC	__	__	setCC>control^*^	setCC>neg control^*^

* *Statistically significant by two-way ANOVA tests and confirmed by Tukey test *(P<0.001)

** Statistically significant by two-way ANOVA tests and confirmed by Tukey test (P<0.05)* †Cold ceramic, ‡Intermediate Restorative Material, # Mineral Trioxide Aggregate* ^^^^ *negative control *

**Table 2:** Mean (SD) values for three subgroups A, B and C

CV^*^	A (1 hr)	B (24 hrs)	C (7 days)
***f *** ^**^ **IRM**	0.49 (0.13)	0.09 (0.07)	0.00 (0.02)
***s***^≠^ **IRM **	0.40 (0.20)	0.20 (0.07)	0.00 (0.02)
***f *MTA**	1.03 (0.12)	0.73 (0.11)	0.47 (0.11)
***s*MTA **	0.93 (0.12)	0.99 (0.10)	0.60 (0.22)
***f*CC**	0.94 (0.24)	0.21 (0.073)	0.02(0.008)
***s*CC**	0.99 (0.32)	0.50 (0.01)	0.41 (0.08)

* Cell viability values according to Elisa Reader, ^**^ *Fresh, ≠Set*

Ghoddusi *et al.* examined the cytotoxicity of MTA and a new endodontic cement using MTT assay and found greater cytotoxicity in the fresh form as well as significant difference among some of their time intervals (24, 48 and 72 hours), concurring with our findings (24). The presence of calcium hydroxide produced as a by-product of the hydration reaction of MTA has been demonstrated (11) whether CC has similar by products is as yet to be determined. The cell viability decrease could be the result of the cell death caused by hydroxyl ion release (high pH) of MTA and possible CC. Freshly mixed MTA causes denaturation of adjacent cells and medium proteins with a surrounding zone of lysed cells. Normal cells can be seen beyond the lyses zone. As the MTA sets, the pH changes and the cell injuries subside (34,35). Notably, studies performed on GICs biocompatibility have been inconclusive. This may be due to the little importance given to the chemical reactions of the material during and after setting (36).

This new prototype material though based on tricalcium silicate; contained admixtures that interfered with the production of calcium hydroxide. Since calcium ion and calcium hydroxide production was shown to be suppressed, the carbonation was less marked. The biocompatibility of the prototype and its variants was similar to that of an established glass-ionomer material (36,34). Cold ceramic however, does not have this disadvantage.

In this study, MTA and CC demonstrated significantly greater cytotoxicity at 7 days compared to one hour (Table 2), possibly due to the gradual release of hydroxyl ion (high alkalinity) over time, which may be neutralized in contact with body tissue fluid but not in *ex vivo* condition. However in set state CC equaled MTA, demonstrating its potential as a root-end filling material in terms of low cytotoxicity.

IRM, though an FDA approved material, had significantly greater cytotoxicity than MTA, a well known biocompatible material (31) as well as CC in almost all subgroups of this study.

A clinical trial demonstrated differences in success rates between IRM and MTA as root-end filling materials (37), supporting previous research (10). However, statistical difference was not established.

## CONCLUSION

Further *in vivo *and *ex vivo *studies are required to assess the properties and clinical use of Cold Ceramic. Our study demonstrated that CC is a competitive material with low cytotoxicity. Cold ceramic may be clinically relevant root-end filling material; having superior cell viability to IRM, a traditional root-end filling material.
